# Insulin resistance and adiponectin levels are associated with height catch-up growth in pre-pubertal Chinese individuals born small for gestational age

**DOI:** 10.1186/1743-7075-9-107

**Published:** 2012-11-28

**Authors:** Hong-Zhu Deng, Hong Deng, Zhe Su, Yan-Hong Li, Hua-Mei Ma, Hong-Shan Chen, Min-Lian Du

**Affiliations:** 1 Department of Pediatrics, The Third Affiliated Hospital of Sun Yat-sen University, Guangzhou, China; 2 Department of Pediatrics, The First Affiliated Hospital of Sun Yat-sen University, Guangzhou, China; 3 Department of Infectious Diseases, The Third Affiliated Hospital of Sun Yat-sen University, Guangzhou, China

**Keywords:** Small for gestational age, Catch-up growth, Insulin resistance, Adiponectin

## Abstract

**Abstracts:**

**Background and objective**

The study was performed to determine whether catch-up growth is associated with the development of insulin resistance and to explore serum endocrine markers associated with the metabolism of adipose tissue in a Chinese population born small for gestational age(SGA)

**Subjects and methods**

We recruited 56 children born SGA with catch-up growth and 55 born without catch-up growth, who were further grouped into groups I (with BMI catch-up) and II (without BMI catch-up) respectively, as well as 52 children born appropriate for gestational age (AGA) with normal height. Their serum fasting insulin, fasting glucose, insulin-like growth factor-1, adiponectin, IGFBP-1, triglyceride concentrations, and the homeostasis assessment model for insulin resistance (HOMA-IR) were evaluated.

**Results**

(1) The HOMA-IR values in SGA-I with catch-up growth group were significantly higher than those in SGA-II with catch-up growth, SGA-I without catch-up growth and AGA children respectively. (2) The serum adiponectin levels of individuals in the SGA-I without catch-up growth and SGA-II with catch-up growth groups were significantly lower than those from the SGA-II without catch-up growth group. There was no difference in triglyceride or IGFBP-1 levels among the groups. (3) The degree of HOMA-IR was positively correlated with age, current BMI and △height SDS in SGA children.

**Conclusion**

The development of insulin resistance and lower levels of adiponectin were closely correlated with higher BMI and the postnatal height catch-up growth in SGA children.

## Introduction

Epidemiological studies have suggested that children born small for gestational age (SGA) are at higher risk for developing metabolic syndromes in adulthood, including insulin resistance, type 2 diabetes, and cardiovascular diseases [1-3]. The “thrifty phenotype” hypothesis suggests that early life metabolic adaptations aid in the survival of an organism by selecting an appropriate growth trajectory in response to adverse intrauterine environmental cues [2]. Catch-up growth in postnatal development is very common in young children born SGA. Previous studies have shown that children born SGA with catch-up growth exhibit fat accumulation, abnormal development of adipose tissue and adipocyte function caused by adverse intrauterine growth, which contributes to the development of insulin resistance [4-7]. However, most of these previous studies did not discuss the contribution of height and fat catch-up growth to insulin resistance, though the strong association between low birth weight and insulin resistance has been described [8-10]. Hence, the discovery of the relative contribution of catch-up growth to the development of insulin resistance is of great importance for developing strategies to prevent insulin resistance-related metabolic syndromes, especially in the Chinese population. Adiponectin, IGFBP-1and triglycerides have been implicated in the pathophysiology of obesity-related insulin resistance, glucose intolerance, and insulin-mediated lipoprotein metabolism. Hypoadiponectinemia has been associated with insulin resistance in both animal and human studies. Only limited and controversial information is available about circulating adiponectin, IGFBP-1and triglyceride levels in pre-pubertal children born SGA [11-13].

This study is a retrospective study aiming to determine whether children born SGA with catch-up growth are associated with the early development of insulin resistance in a Chinese population and investigating adiponectin, IGFBP-1and triglyceride levels in SGA children, while accounting for the height and fat catch-up growth.

## Subjects and methods

A total of 111 children born SGA and 52 children born AGA were recruited from out-patient services at the Department of Pediatrics and Obstetrics of the First Affiliated Hospital, Sun Yat-Sen University for regular physical examination from Jan 2006 to Dec 2009. The SGA children were all follow-up cases born in our hospital, and they received regular physical examination once every one to three months. The maternal age of SGA groups was (30 ± 4) years. Multiple gestation(30/111, 27%), gestational hypertension/pre-eclampsia (21/111, 19%), pre-existing (before pregnancy) diabetes(12/111,11%), gestational diabetes (10/111, 9%), intrahepatic cholestasis of pregnancy(8/111, 7%), maternal infection with syphilis or HIV(3/111, 3%), maternal smoking or alcohol use or illicit drug use during pregnancy(2/111, 2%) were included in SGA groups.

All of the children with age range 3.5yr to 10.2yr were born between 37 and 42 weeks of gestation, were non-obese (based on the standard deviation score (SDS) of body mass index ≤2 for the chronological age and gender) and were in the pre-pubertal period defined as Tanner I. SGA was defined as a birth weight and length < −2 standard deviation (SD), and AGA was defined as individuals with a birth weight and length ≥−2 SD and ≤ 2 SD for the gestational age, according to Chinese standards [9]. The SGA children were further divided into four groups (Table 1): ① SGA-I with catch-up growth (*n*=40; 23F, 17M), ② SGA-II with catch-up growth (*n*=16; 7F, 9M), ③ SGA-I without catch-up growth (*n*=37; 16F, 21M),and ④ SGA-II without catch-up growth (*n*=18; 8F, 10M). Children born SGA without catch-up growth had serum maximum growth hormone (GH) levels of greater than 10 μg/L(results from GH-provocative tests using Levodopa and Pyridostigmine). GH therapy was not administered to all of the SGA Children during the follow-up period. The AGA group of children (*n*=52; 23F, 29M) with normal height and weight were matched according to age, gender and Tanner stage in the SGA groups.

**Table 1:** Grouping criteria for the SGA and AGA groups

**①SGA-I with catch-up growth (*n*=40; 23F, 17M)**	Birth length SDS< −2, Height SDS_Corrected_>−2 and △BMI SDS>0.
**②SGA-II with catch-up growth (*n*=16; 7F, 9M)**	Birth length SDS< −2, Height SDS_Corrected_>−2 and △BMI SDS≤0
**③SGA-I without catch-up growth (*n*=37; 16F, 21M)**	Birth length SDS< −2, Height SDS_Corrected_≤−2 and △Height SDS≤0 and △BMI SDS>0
**④SGA-II without catch-up growth (*n*=18; 8F, 10M)**	Birth length SDS< −2, Height SDS_Corrected_≤−2 and △Height SDS≤0 and △BMI SDS≤0
**⑤AGA(*n*=52; 23F, 29M)**	Birth length SDS≥ −2 and ≤2, Height SDS_Corrected_>−2

AGA: Appropriate for gestational age; SGA: Small for gestational age; BMI: Body mass index; Height SDS_Corrected_: Actual height SDS minus target height SDS; △Height SDS: Current height SDS corrected minus birth length SDS; △BMI SDS: Current BMI SDS minus birth BMI SDS.

Individual subjects who had chromosome abnormalities, congenital skeletal deformations, hypothyroidism, congenital malformation-related growth retardation, chronic diseases, or long-term medication were excluded. Children, with first-degree relatives who suffered from type 2 diabetes and metabolic syndrome, or who were born from mothers with gestational diabetes, were excluded. Obese children were also excluded to avoid another confounding factor in our study.

Informed consent was obtained from their parents, and the experimental 1 protocol was approved by the Local Ethics Committee.

### Measurements

An individual’s birth length and weight were obtained from hospital records and their target heights were calculated as mid-parental height minus 6.5 cm for girls and mid-parental height plus 6.5 cm for boys. Their current height, weight, and body mass index (BMI) were also measured. Age- and gender-adjusted SDS for height, weight, and BMI were calculated using reference tables of Chinese children [14,15]. The SDScorrected height for individual subjects was calculated using the actual height SDS minus target height SDS. The △ height SDS was calculated using the current height SDScorrected minus birth length SDS, while the △weight SDS was determined using the current weight SDS minus birth weight SDS. The BMI of individual children was calculated as weight (kg)/height (m)^2^ . The △BMI SDS was determined using the current BMI SDS minus birth BMI SDS. The demographic characteristics and measured values are listed in Table 2.

**Table 2:** Clinical and anthropometric parameters of SGA and AGA children at birth and investigation

	**SGA-I with catch-up growth**	**SGA-II with catch-up growth**	**SGA-I without catch-up growth**	**SGA-II without catch-up growth**	**AGA**
	***n*=40**	***n*=16**	***n*=37**	***n*=18**	***n*=52**
Gender(M:F)	17:23	9:7	21:16	10:8	29:23
Midparental height (cm)	161.38±0.80	162.27±0.45	162.75±0.54	162.00±0.73	160.14±0.44
Age(year)	6.78±0.88	6.27±0.96	5.30±0.67	5.91±0.98	7.14±0.39
Gestational age (weeks)	38.76±0.21	39.17±0.19	38.76±0.21	39.17±0.19	39.23±0.14
Birth weight SDS	−2.27±0.18^*^	−1.82±0.39^*^	−2.61±0.11^*^	−1.83±0.25^*^	0.23±0.04
Birth length SDS	−2.42±0.14^*^	−3.00±0.25^*^	−2.02±0.21^*^	−2.29±0.19^*^	0.14±0.03
Birth BMI SDS	−1.51±0.37^*^	−1.29±0.45^*^	−1.91±0.45^*^	−1.47±0.38^*^	0.39±0.24
Weight SDS	−1.65±0.41	−2.18±0.53	−2.11±0.37	−4.02±0.57^*^	−1.41±0.25
Height SDS_Corrected_	−0.93±0.22	−1.49±0.15	−3.24±0.18^*^	−2.82±0.29^*^	−1.05±0.07
BMI SDS	−0.15±0.19 ^#^	−1.18±0.19	−0.76±0.25 ^#^	−1.63±0.25^*^	−0.32±0.12
△Height SDS	1.57±0.27^*^	1.51±0.21^*^	−1.24±0.26	−1.21±0.23	−0.73±0.28
△Weight SDS	0.63±0.39^* #^	−0.36±0.34	0.50±0.35^* #^	−2.19±0.46	−1.52±0.37
△BMI SDS	1.36±0.47^*^^#^	−0.06±0.35	1.33±0.36^* #^	−0.17±0.34	−0.71±0.25

*P<0.05 SGA-I or SGA-II vs AGA; ^#^P<0.05 SGA-I vs SGA-II.

AGA: Appropriate for gestational age; SGA: Small for gestational age; BMI: Body mass index; △Height SDS: Current height SDS corrected minus birth length SDS; △Weight SDS: Current Weight SDS minus birth Weight SDS; △BMI SDS: Current BMI SDS minus birth BMI SDS.

### Laboratory examinations

After overnight fasting, blood samples were collected and sera were prepared by centrifugation and stored at −70°C. The serum glucose concentrations were analysed immediately. The concentration of serum insulin and IGF-I for individual subjects were simultaneously determined via routine laboratory examinations. The concentration of blood glucose was determined using the hyperoxidase method (Photometric Instrument 4010; Roche, Basel, Switzerland). The intra-assay coefficient of variation of this method was <2.0%. The serum insulin concentrations were determined via radioimmunoassay (RIA) using the insulin RIA kit, according to the manufacturer’s instructions (Phadeseph Insulin RIA, Pharmacia &Upjohn Diagnostics AB, Uppsala, Sweden). The intra- and inter-assay coefficients of variation were 5.3% and 7.6%, respectively. The serum IGF-I concentrations were determined using an ELISA kit, according to the manufacturer’s instructions (Diagnostic Systems Laboratories −10-2800, Inc., Webster, TX, USA). The sensitivity of the assay was 0.01 ng/mL. The inter- and intra-assay coefficients of variation were 3.3-6.8% and 4.5-8.6%, respectively. The HOMA-IR was calculated as insulin (microunits per millilitre) × glucose (millimoles per litre) /22.5 and was used to evaluate IR in individual children [16].

### Statistical analyses

Data were analysed using the Statistical Package for Social Sciences (SPSS, version 13.0 SPSS Inc, Chicago, IL, USA). Data for HOMA-IR were transformed into normal distributions by calculating the natural logarithms and were expressed as the geometric mean ± standard error of mean (SEM). All other values were expressed as the arithmetic mean ±SEM. Comparisons among the groups were performed using parametric tests. The differences among groups were assessed by one-way ANOVA and univariate analysis using Fisher’s protected least significant difference test for inter-group comparisons. The differences between two groups were determined using unpaired *t* tests. Differences in gender distribution between groups were assessed by *χ*2 -tests. Relationships between variables were analysed by simple correlation (Pearson’s test) and general linear models. A value of *P*<0.05 was considered statistically significant.

## Results

### Anthropometry

There was no difference in gender, age, or gestational age among these groups. As expected, birth weights and lengths SDS were significantly lower in SGA children than in AGA children. The values of height SDS_corrected_ in SGA children without catch-up growth were lower than those of SGA with catch-up growth and AGA children (*P*<0.001). Moreover the values of BMI SDS in SGA-I with and without catch-up growth children were higher than those of SGA-II with and without catch-up growth children respectively (*P*<0.001). Furthermore, the values of BMI SDS in SGA-II without catch-up growth children were lower than in AGA children (*P=*0.001), but there were no significant difference compared to the children in the SGA-II with catch-up growth group (*P*=0.073). There was no difference in BMI SDS among the SGA-I with and without catch-up growth and AGA groups (*P*>0.05, Table 2).

### Laboratory profile

There was no significant difference in the concentration of fasting serum glucose among the groups of children. The HOMA-IR values in SGA-I children with catch-up growth were significantly higher than in SGA-II with catch-up growth, SGA-I without catch-up growth and AGA children. These differences persisted despite adjusting for gender, age, and BMI (*P*=0.022, 0.015, 0.029, respectively). The HOMA-IR values in two SGA groups without catch-up growth were all comparable to the values in AGA children (*P*>0.05).

The levels of adiponectin in the SGA-II with catch-up growth and SGA-I without catch-up growth groups were significantly lower than those in the SGA-II without catch-up growth group respectively (*P*=0.008, 0.011, respectively). These differences appeared despite adjusting for BMI (*P*=0.038, 0.035, respectively). However, there was no significant difference when compared to the AGA group. There was no significant difference in the IGFBP-1and TG values among those groups even after adjusting for BMI (*P*>0.05) (Table 3).

**Table 3:** Laboratory profile of SGA and AGA children at investigation

	**SGA-I with catch-up growth**	**SGA-II with catch-up growth**	**SGA-I without catch-up growth**	**SGA-II without catch-up growth**	**AGA**
	***n*=40**	***n*=16**	***n*=37**	***n*=18**	***n*=52**
Fasting glucose(mmol/L)	4.55±0.13	4.68±0.14	4.33±0.16	4.66±0.18	4.73±0.07
Fasting insulin(uU/mL)	11.25±3.41^* #▲^	5.10±0.64	3.94±0.67	3.10±0.66	5.39±0.40
HOMA-IR	2.30±0.71^* #▲^	1.04±0.11	0.81±0.15	0.65±0.14	1.15±0.09
Triglyceride(mmol/L)	0.96±0.13	1.03±0.18	1.10±0.12	0.85±0.14	0.86±0.04
Adiponectin(ug/mL)	0.07±0.01	0.09±0.02	0.09±0.01	0.15±0.07^* #▲^	0.09±0.01
IGFBP-1(ng/mL)	111.08±8.75	103.85±15.25	90.18±12.74	123.44±21.15	106.54±11.96
IGF-1(ng/mL)	215.47±19.99	206.56±37.77	126.56±16.35^#▲^	171.11±31.32	175.53±13.69

*P<0.05 SGA-I or SGA-II vs AGA; ^#^P<0.05 SGA-I vs SGA-II; ^#▲^P<0.05 SGA-I with catch-up growth vs SGA-I without catch-up growth; SGA-II with catch-up growth vs SGA-II without catch-up growth.

AGA: Appropriate for gestational age; SGA: Small for gestational age; HOMA: Homeostasis assessment model; IR: Insulin resistance; IGF-1: Insulin-like growth factor-1; IGFBP-1: Insulin-like growth factor binding protein-1.

### Correlation analysis

Correlation analysis indicated that the HOMA-IR values were positively correlated with age(*r*=0.375, *P*=0.003), △Ht SDS(*r*=0.431, *P*=0.000) and BMI (*r*=0.417, *P*=0.001), however, they were not associated with birth weight (*P>0.05*).

The HOMA-IR values were negatively correlated with the adiponectin and IGFBP-1 levels (*r*=−0.33, *P*=0.03; *r*=−0.43, *P*<0.01) in the SGA group, but no correlation was observed with the levels of triglycerides. Adiponectin and IGFBP-1 levels exhibited a negative correlation with chronological age (*r*=−0.427, *P*=0.001; *r*=−0.289, *P*=0.023).

## Discussion

In our study, SGA children, who had made a height catch-up growth accompanied by a BMI catch-up growth, had significantly higher values of insulin resistance index compared to AGA children. This finding showed that the development of insulin resistance was associated with height and BMI catch-up growth in SGA children, which increased with age. These data were not in complete accordance with several previous reports [9,17,18]. The majority of previous studies did not analyse the contribution of BMI catch-up growth to insulin resistance during postnatal catch-up growth, though the strong association between low birth weight and insulin resistance has been described [9,17,19]. In our study population, △BMI SDS values were higher in SGA children than in AGA children. Because the high degree of BMI usually reflects the excess accumulation of fat in the body, our data suggest that there was body fat accumulation in SGA children, especially with catch-up growth in height during childhood and that the excess fat contributes to the development of insulin resistance in SGA children. SGA children with higher current BMI were more insulin-resistant than AGA children, in spite of their similar weight and BMI. The data also suggested that insulin resistance may precede the development of obesity. Other studies have suggested that low birth weight is a risk factor for the later development of abdominal or truncal obesity, and SGA children with catch-up weight gain show a dramatic transition toward central adiposity, which enhances insulin resistance [20-23]. Accordingly, the measures used to control overweight when gaining linear catch-up growth in childhood seem to be important in the prevention of insulin resistance in SGA children. Our findings indicated that height catch-up growth was an important influential factor for insulin resistance in SGA children. Our work might contribute to understanding the involvement of catch-up growth in the pathogenesis of insulin resistance in SGA groups. The implications of our results in relation to catch-up in height might be of potential importance when considering GH treatment.

Decreased adiponectin and IGFBP-1 levels and increased triglyceride levels are considered to reflect impaired insulin sensitivity and predict insulin resistance. Data on adiponectin, IGFBP-1 and triglyceride levels in SGA children in the literature have varied [24-27]. Adiponectin is one of the adipokines produced exclusively by adipocytes. Adiponectin knockout mice develop glucose intolerance, insulin resistance, and hyperlipidemia, especially when fed high-fat diets [28]. Decreased levels of plasma adiponectin have been found to be related to obesity, type 2 diabetes, and cardiovascular disease. Cianfarani *et al.* reported that the serum adiponectin levels were lower in SGA children (aged 8.6±3.5yr) than in short-normal children born AGA [29]. These differences were not observed in the research of Lopez-Bermejo *et al.* whose extensive analysis revealed that the group of overweight SGA children had lower serum adiponectin concentrations than lean SGA children [30]. Research on preterm infants had shown that the change in the serum adiponectin levels closely correlated to gains in body weight in AGA and SGA children [31]. One important finding of the present study was that adiponectin was not only associated with BMI catch-up, but also with linear catch-up growth in pre-pubertal SGA children. Our study also showed that adiponectin was inversely associated with insulin resistance markers. The results highlight that BMI and linear catch-up might be two independent determinants of hypoadiponectinemia in SGA children. Previous studies have shown that low birth weight followed by catch-up in body fat, especially visceral fat during childhood, even within the normal weight range, was associated with a higher risk of developing insulin resistance [20,22]. Our study also showed that the decreased adiponectin levels were associated with postnatal body fat accumulation in SGA children and added new information on this association because it showed the relevant role of fat and height catch-up. To the best of our knowledge, the relationship between linear catch-up and adiponectin are novel and may contribute to understanding the involvement of adiponectin in the pathogenesis of insulin resistance in SGA children.

IGFBP-1, produced in the liver, is suppressed by insulin through binding to insulin-response elements in the IGFBP-1 gene promoter, which forms a link between glucose metabolism and IGF axis [32]. Insulin resistance and impaired glucose tolerance are observed in IGFBP-1 transgenic mice [33]. Reduced serum IGFBP-1 levels are considered to reflect hyperinsulinemia and cardiovascular risk in adults and obese children [32,34]. Kamoda T *et al.* reported that the levels of serum IGFBP-1 were similar in short children born SGA and AGA [35]. Kistner A *et al.* investigated the serum markers of insulin resistance in adults born SGA and reported that lower IGFBP-1 and triglyceride levels were observed in the SGA group compared to the AGA group despite with normal BMI [36]. In our study, there were no marked differences in IGFBP-1 and triglyceride levels between SGA children with height and BMI catch-up growth and their age-matched controls. In SGA children, especially non-catch-up growth subjects, the lower BMI may have influenced IGFBP-1and triglyceride levels, although we had adjusted for BMI. Further analysis showed that IGFBP-1 correlated significantly with the HOMA-IR. values. These differences and correlations were not observed with triglyceride levels. In this instance, our results were similar to the results published by Evagelidou EN *et al.*, who reported that SGA children, although more insulin-resistant, had similar triglyceride levels to AGA children in pre-puberty [37]. Our findings indicated that adiponectin might be a more effective marker than IGFBP-1 and triglyceride for monitoring insulin resistance in SGA children with catch-up growth. However, these three markers were not sensitive enough at this age stage in non-obese SGA children.

We need to be aware of the limitations in interpreting the results of this study. The major limitation of the present study is the relatively small sample size. Another limitation is that the study design was based on retrospective data collecting. The third limitation is that both body fat and insulin resistance were studied using surrogate variables. These limitations might suggest that the results are not easily applicable to the whole population of pre-pubertal SGA children. However, we have presented the presence of four different SGA populations compared to normal controls in the exploration of system-wide adiponectin, IGFBP-1, triglyceride levels and insulin resistance in SGA children, while considering height and fat catch-up growth.

In conclusion, our study showed that children born SGA with catch-up growth for height and BMI are more insulin-resistant than children born AGA. Our study indicated that interventions to control overweight development in childhood seem to be important for preventing insulin resistance. Furthermore, lower levels of adiponectin were closely correlated with height and BMI catch-up growth in SGA children. Adiponectin may be a more useful marker than IGFBP-1 and triglyceride for monitoring insulin resistance in SGA children with catch-up growth.

## Abbreviations

AGA: Appropriate for gestational age; SGA: Small for gestational age; BMI: Body mass index; SD: Standard deviation; SDS: Standard deviation score(s); IGF-1: Insulin-like growth factor-1; GH: Growth hormone; HOMA: Homeostasis assessment model; IR: Insulin resistance; IGFBP-1: Insulin-like growth factor binding protein-1.

## Competing interests

The authors have nothing to disclose.

## Authors’ contributions

HZD participated in the study design, execution and analysis, and also drafted the manuscript. HD participated in the execution, data analysis and interpretation of the study, and contributed to manuscript preparation. ZS participated in collection cases. YHL participated in collection cases. HMM participated in collection cases. HSC participated in collection cases. MLD participated in study design and data analysis, and contributed to manuscript preparation. All authors read and approved the final manuscript.

## References

[B1] Varvarigou AA Intrauterine growth restriction as a potential risk factor for disease onset in adulthood J Pediatr Endocrinol Metab 2010 23 215 224 20480719 10.1515/jpem.2010.23.3.215

[B2] Hofman PL Cutfield WS Robinson EM Bergman RN Menon RK Sperling MA Gluckman PD Insulin resistance in short children with intrauterine growth retardation J Clin Endocrinol Metab 1997 82 402 406 10.1210/jc.82.2.402 9024226

[B3] Rotteveel J van Weissenbruch MM Twisk JW Delemarre-Van de Waal HA Infant and childhood growth patterns, insulin sensitivity, and blood pressure in prematurely born young adults Pediatrics 2008 122 313 321 10.1542/peds.2007-2012 18676549

[B4] Fabricius-Bjerre S Jensen RB Færch K Larsen T Mølgaard C Michaelsen KF Vaag A Greisen G Impact of birth weight and early infant weight gain on insulin resistance and associated cardiovascular risk factors in adolescence PLoS One 2011 6 e20595 10.1371/journal.pone.0020595 21655104 PMC3107215

[B5] Maiorana A Del Bianco C Cianfarani S Adipose tissue: a metabolic regulator Potential implications for the metabolic outcome of subjects born small for gestational age (SGA) Rev Diabet Stud 2007 4 134 146 10.1900/RDS.2007.4.134 18084671 PMC2174062

[B6] Ong KK Ahmed ML Emmett PM Preece MA Dunger DB Association between postnatal catch-up growth and obesity in childhood: prospective cohort study BMJ 2000 320 967 971 10.1136/bmj.320.7240.967 10753147 PMC27335

[B7] Jaquet D Deghmoun S Chevenne D Collin D Czernichow P Lévy-Marchal C Dynamic change in adiposity from fetal to postnatal life is involved in the metabolic syndrome associated with reduced fetal growth Diabetologia 2005 48 849 855 10.1007/s00125-005-1724-4 15834547

[B8] Dallar Y Dilli D Bostanci I Oğüş E Doğankoç S Tuğ E Insulin sensitivity obtained from the oral glucose tolerance test and its relationship with birthweight Ann Saudi Med 2007 27 13 17 10.4103/0256-4947.51496 17582913 PMC6077022

[B9] Veening MA Van Weissenbruch MM Delemarre-Van De Waal HA Glucose tolerance, insulin sensitivity, and insulin secretion in children born small for gestational age J Clin Endocrinol Metab 2002 87 4657 4661 10.1210/jc.2001-011940 12364453

[B10] Veening MA van Weissenbruch MM Heine RJ Delemarre-van de Waal HA Beta-cell capacity and insulin sensitivity in prepubertal children born small for gestational age: influence of body size during childhood Diabetes 2003 52 1756 1760 10.2337/diabetes.52.7.1756 12829643

[B11] Lihn AS Pederson SB Richeksen B Adiponectin: action, regulation and association to insulin sensitivity Obes Rev 2005 6 13 21 10.1111/j.1467-789X.2005.00159.x 15655035

[B12] Mogul HR Marshall M Frey M Burke HB Wynn PS Wilker S Southern AL Gambert SR Insulin like growth factor-binding protein-1 as a marker for hyperinsulinaemia in obese menopausal women J Clin Endocrinol Metab 1996 81 4492 4495 10.1210/jc.81.12.4492 8954066

[B13] Jones JN Gercel-Taylor C Taylor DD Altered cord serum lipid levels associated with small for gestational age infants Obstet Gynecol 1999 93 527 531 10.1016/S0029-7844(98)00489-X 10214827

[B14] Zhang BL Feng ZK Zhang LH Birth size standards by gestational age for Chinese neonates Zhonghua Er Ke Za Zhi 1988 26 206 208 in Chinese

[B15] Hu YM Jiang ZF Growth development standards for Chinese children Zhu FuTang practical pediatr 2002 7 BeiJing: The People Health press 27 34 in Chinese

[B16] Matthews DR Hosker JP Rudenski AS Naylor BA Treacher DF Turner RC Homeostasis model assessment: insulin resistance and β-cell function from fasting plasma glucose and insulin concentrations in man Diabetologia 1985 28 412 419 10.1007/BF00280883 3899825

[B17] Soto N Bazaes RA Peña V Salazar T Avila A Iñiguez G Ong KK Dunger DB Mericq MV Insulin sensitivity and secretion are related to catch-up growth in small-for- gestational-age infants at age 1 year: results from a prospective cohort J Clin Endocrinol Metab 2003 88 3645 3650 10.1210/jc.2002-030031 12915649

[B18] Deng HZ Li YH Su Z Ma HM Huang YF Chen HS Du ML Association between height and weight catch-up growth with insulin resistance in pre-pubertal Chinese children born small for gestational age at two different ages Eur J Pediatr 2011 170 75 80 10.1007/s00431-010-1274-8 20734204

[B19] Iñiguez G Ong K Bazaes R Avila A Salazar T Dunger D Mericq V Longitudinal changes in insulin-like growth factor-1, insulin sensitivity, and secretion from birth to age three years in small-for-gestational-age children J Clin Endocrinol Metab 2006 91 4645 4649 10.1210/jc.2006-0844 16912131

[B20] Dulloo AG Regulation of fat storage via suppressed thermogenesis: a thrifty phenotype that predisposes individuals with catch-up growth to insulin resistance and obesity Horm Res 2006 65 90 97 10.1159/000091512 16612120

[B21] Ibáñez L Ong K Dunger DB de Zegher F Early development of adiposity and insulin resistance after catch-up weight gain in small-for-gestational-age children J Clin Endocrinol Metab 2006 91 2153 2158 10.1210/jc.2005-2778 16537681

[B22] Ibáñez L Suárez L Lopez-Bermejo A Díaz M Valls C de Zegher F Early development of visceral fat excess following spontaneous catch-up growth in children with low birthweight J Clin Endocrinol Metab 2008 93 925 928 18089700 10.1210/jc.2007-1618

[B23] Leunissen RW Oosterbeek P Hol LK Hellingman AA Stijnen T Hokken-Koelega AC Fat mass accumulation during childhood determines insulin sensitivity in early adulthood J Clin Endocrinol Metab 2008 93 445 451 10.1210/jc.2007-1543 18042649

[B24] Sancakli O Darendeliler F Bas F Gokcay G Disci R Aki S Eskiyurt N Insulin, adiponectin, IGFBP-1 levels and body composition in small for gestational age born non-obese children during prepubertal ages Clin Endocrinol (Oxf) 2008 69 88 92 10.1111/j.1365-2265.2007.03138.x 18031314

[B25] Ibáñez L Lopez-Bermejo A Diaz M de Zegher F Catch-up growth in girls born small for gestational age precedes childhood progression to high adiposity Fertil Steril 2011 96 220 223 10.1016/j.fertnstert.2011.03.107 21549368

[B26] Miras M Ochetti M Martín S Silvano L Sobrero G Castro L Onassis M Tolosa de Talamoni N Pérez A Picotto G Díaz de Barboza G Muñoz L Serum levels of adiponectin and leptin in children born small for gestational age: relation to insulin sensitivity parameters J Pediatr Endocrinol Metab 2010 23 463 471 20662345 10.1515/jpem.2010.077

[B27] Challa AS Evagelidou EN Cholevas VI Kiortsis DN Giapros VI Drougia AA Andronikou SK Growth factors and adipocytokines in pre-pubertal children born small-for-gestational age: relation to insulin resistance Diabetes Care 2009 32 714 719 10.2337/dc08-1570 19131467 PMC2660477

[B28] Blackburn GL From bench to bedside: novel mechanisms and therapeutic advances through the development of selective peroxisome proliferator-activated receptor c modulators Am J Clin Nutr 2010 91 251S 253S 10.3945/ajcn.2009.28449A 19923371

[B29] Cianfarani S Martinez C Maiorana A Scirè G Spadoni GL Boemi S Adiponectin levels are reduced in children born small for gestational age and are inversely related to postnatal catch-up growth J Clin Endocrinol Metab 2004 89 1346 1351 10.1210/jc.2003-031704 15001632

[B30] López-Bermejo A Casano-Sancho P Fernández-Real JM Kihara S Funahashi T Rodríguez-Hierro F Ricart W Ibañez L Both intrauterine growth restriction and postnatal growth influence childhood serum concentrations of adiponectin Clin Endocrinol (Oxf) 2004 61 339 346 10.1111/j.1365-2265.2004.02102.x 15355450

[B31] Saito M Nishimura K Nozue H Miyazono Y Kamoda T Changes in serum adiponectin levels from birth to term-equivalent age are associated with postnatal weight gain in preterm infants Neonatology 2011 100 93 98 10.1159/000322654 21273794

[B32] van der Kaay D Deal C de Kort S Willemsen R Leunissen R Ester W Paquette J van Doorn J Hokken-Koelega A Insulin-like growth factor-binding protein-1: serum levels, promoter polymorphism, and associations with components of the metabolic syndrome in short subjects born small for gestational age J Clin Endocrinol Metab 2009 94 1386 1392 10.1210/jc.2008-1430 19158202

[B33] Murphy LJ Over expression of insulin-like growth factor binding protein-1 in transgenic mice Pediatr Nephrol 2000 14 567 571 10.1007/s004670000347 10912520

[B34] Gokulakrishnan K Velmurugan K Ganesan S Mohan V Circulating levels of insulin-like growth factor binding protein-1 in relation to insulin resistance, type 2 diabetes mellitus, and metabolic syndrome (Chennai Urban Rural Epidemiology Study 118) Metabolism 2012 61 43 46 10.1016/j.metabol.2011.05.014 21741060

[B35] Kamoda T Nozue H Matsui A Serum levels of adiponectin and IGFBP-1 in short children born small for gestational age Clin Endocrinol (Oxf) 2007 66 290 294 10.1111/j.1365-2265.2006.02724.x 17224001

[B36] Kistner A Jacobson SH Celsi G Vanpee M Brismar K IGFBP-1 levels in adult women born small for gestational age suggest insulin resistance in spite of normal BMI J Intern Med 2004 255 82 88 10.1046/j.0954-6820.2003.01257.x 14687242

[B37] Evagelidou EN Giapros VI Challa AS Kiortsis DN Tsatsoulis AA Andronikou SK Serum adiponectin levels, insulin resistance, and lipid profile in children born small for gestational age are affected by the severity of growth retardation at birth Eur J Endocrinol 2007 156 271 277 10.1530/eje.1.02337 17287418

